# Performance Profiles in Youth Basketball Across Different Score Contexts: An Unsupervised Machine Learning Analysis

**DOI:** 10.3390/jfmk11030282

**Published:** 2026-07-21

**Authors:** Dimitrios Pantazis, Christos Kokkotis, Alexandra Avloniti, Theodoros Stampoulis, Panagiotis Foteinakis, Panagiotis Aggelakis, Dimitrios Balampanos, Maria Protopapa, Alexandros Dendrinos, Konstantinos Margonis, Nikolaos Zaras, Georgios Pafis, Paraskevi Malliou, Maria Michalopoulou, Athanasios Chatzinikolaou

**Affiliations:** 1 Department of Physical Education and Sport Science, School of Physical Education, Sport Science and Occupational Therapy, Democritus University of Thrace, 69100 Komotini, Greece; dpantazi@phyed.duth.gr (D.P.); alavloni@phyed.duth.gr (A.A.); pfotinak@phyed.duth.gr (P.F.); pangelak@phyed.duth.gr (P.A.); dimibala10@phyed.duth.gr (D.B.); mprotopa@phyed.duth.gr (M.P.); adendrin@phyed.duth.gr (A.D.); kmargoni@phyed.duth.gr (K.M.); nzaras@phyed.duth.gr (N.Z.); gpafis@phyed.duth.gr (G.P.); pmalliou@phyed.duth.gr (P.M.); michal@phyed.duth.gr (M.M.); 2 Department of Occupational Therapy, School of Physical Education, Sport Science and Occupational Therapy, Democritus University of Thrace, 69100 Komotini, Greece; ckokkoti@ot.duth.gr (C.K.); tstampou@ot.duth.gr (T.S.)

**Keywords:** basketball performance analysis, unsupervised machine learning, k-means clustering, physical load, movement intensity, offensive performance

## Abstract

**Objectives**: The analysis of basketball performance has increasingly incorporated advanced analytics and machine learning methods to better understand the factors that influence offensive efficiency and match dynamics. The present study aimed to identify performance profiles in basketball using unsupervised machine learning techniques and to examine the physical load and performance indicators that differentiate these profiles. **Methods**: Team-quarter observations from the Final 8 phase of the Greek U16 Basketball Championship were stratified into quarters with large score differences and quarters with small score differences according to the quarter-specific score differential and the sample median of 5 points. K-means clustering was applied separately to each dataset to identify latent performance patterns. Candidate solutions were evaluated using the Elbow method, Silhouette coefficient, Calinski–Harabasz Index, and Davies–Bouldin Index. Based on their combined interpretation, together with considerations of parsimony and practical interpretability, two-cluster solutions were retained for both datasets. Cluster stability was assessed using the Adjusted Rand Index (ARI), while t-distributed stochastic neighbor embedding (t-SNE) was used exclusively for visualization of the identified clusters. **Results**: Welch’s independent-samples *t*-tests with Benjamini–Hochberg false discovery rate (FDR) correction identified significant differences between clusters across several external load variables, including jump load, total distance covered, accumulated acceleration load, and distance covered in different speed zones (pFDR < 0.001). Clusters characterized by higher movement intensity also exhibited higher values for basketball performance and offensive-efficiency indicators. Although higher-performance clusters showed numerically higher winning proportions in both contexts (large score differences: 70.0% vs. 45.7%; small score differences: 56.7% vs. 40.6%), chi-square analyses indicated that cluster membership was not significantly associated with quarter outcomes. **Conclusions**: Overall, the findings suggest that performance profiles in basketball are primarily differentiated by external-load characteristics, particularly movement intensity, and offensive-performance indicators, highlighting the importance of integrating both physical and technical performance indicators in basketball performance analysis.

## 1. Introduction

Performance analysis has become an essential component of team sports, enabling coaches and performance staff to obtain objective, systematic match information to inform decisions concerning game planning, performance evaluation, and load management [1,2,3,4,5,6,7]. In this context, team sports are increasingly conceptualized as complex, dynamic systems composed of multiple interacting elements in which players and teams continuously adapt to one another and to environmental constraints [6,8,9,10,11]. Nonlinear interactions, instability, and sensitivity to contextual factors distinguish such systems. Accordingly, performance outcomes arise from interactions among multiple factors rather than from any single variable. This perspective shifts performance analysis from exclusively outcome-based approaches toward more holistic frameworks [5,6,12].

Within this framework, basketball is a highly complex and intermittent team sport in which performance emerges from the continuous interaction among technical, tactical, and physical components [13]. Conventional box-score statistics, including points, rebounds, assists, steals, turnovers, and shooting efficiency, have long been used to evaluate individual and team contributions and to identify factors associated with match outcomes [14,15,16,17,18]. Meanwhile, physical performance is expressed through high-intensity locomotor actions, including accelerations, decelerations, changes of direction, and jumping events, collectively defining the sport’s external load demands [19,20,21]. Basketball performance is also influenced by contextual factors. These factors can affect external load, defined as the physical demands imposed on the athlete, and internal load, which reflects the psychophysiological response to those demands [22]. They may also influence match-related statistics reflecting technical and tactical efficiency [16,17,18,23,24]. Among these factors, score differential and match outcome represent important contextual constraints that shape match dynamics [25]. Specifically, it has been demonstrated that balanced match situations, typically characterized by minimal score margins, are associated with greater external loads than imbalanced match situations, where teams may regulate their effort and the pace of the match [22].

Basketball matches consist of consecutive quarters separated by rest intervals and coaching interventions. The physical, technical, and tactical demands of one quarter may influence player behavior and performance in subsequent quarters through fatigue accumulation, pacing, score evolution, and tactical adjustments [8,25,26]. Consequently, analyzing each quarter seperately may allow more precise characterization of successful and unsuccessful periods of play, as contextual dynamics and performance indicators can vary substantially across match quarters despite similar final match outcomes [27,28]. For instance, external demands appear to be largely consistent across different score margins, with only minor increases observed during quarters exhibiting larger point differences [29]. Similarly, internal load metrics, including session rating of perceived exertion (sRPE) and summated heart rate zones (SHRz), show elevated values under balanced conditions, reflecting the heightened physiological demands and competitive characteristics of close-score situations [22]. However, contrasting findings have been reported, with studies showing no significant influence of score differential on perceived exertion, suggesting that contextual factors may depend on competition level and sex [25,30].

Although several studies have examined contextual factors and performance responses in professional and semi-professional basketball, and more recently in under-18 players, evidence in younger age categories, such as U16, remains limited [29,31,32,33]. Youth basketball presents distinct performance characteristics compared to senior levels, largely due to differences in physical development, training exposure, and tactical maturity [29,34,35]. Recent research [29,35] has indicated that, in contrast to findings in adult populations [22], no significant disparities were identified between winning and losing quarters in youth basketball across most external load variables. However, further analysis reveals that contextual factors, such as the score differential, appear to exert a more significant effect. Specifically, among losing quarters, close-score situations were associated with greater movement demands, including higher inertial movement analysis and change-of-direction values, than unbalanced situations. No significant differences were observed across score-differential categories among winning quarters, suggesting a more stable performance profile when teams were leading [35].

The analysis of basketball performance has evolved significantly in recent years, integrating advanced analytics and machine learning (ML) methods and moving beyond traditional box-score statistics toward a more nuanced understanding of match dynamics [16,18,23]. The emergence of advanced statistics, such as Performance Index Rating (PIR), effective field goal percentage (eFG%), and offensive and defensive ratings, along with the ability of ML methods to analyze complex, multidimensional data has enabled analysts to characterize team performance more comprehensively [14,36]. Nevertheless, most applications of advanced analytics have been confined to professional leagues, with limited translation to youth contexts where skill variability, tactical discipline, and physical maturation substantially alter performance dynamics. Despite the growing use of performance analytics in basketball, limited research has examined performance patterns using unsupervised ML approaches that integrate both physical load and technical performance indicators. Rather than testing predefined hypotheses, unsupervised machine learning aims to identify latent structures within multidimensional datasets by grouping observations according to their overall similarity. Identifying latent performance structures within match quarters may provide a more comprehensive understanding of how movement intensity and performance efficiency interact within individual match quarters. Therefore, the present study aimed to identify distinct performance profiles in U16 basketball using an unsupervised ML approach integrating external load demands and match-related basketball statistics. The identified clusters were subsequently described according to their external-load and match-related performance characteristics and explored in relation to quarter outcome. To the best of the authors’ knowledge, this is the first study to examine quarter-based external load demands and basketball-related statistics in a national-level U16 basketball competition using a data-driven analytical framework. Specifically, the study sought to determine whether match-quarter performance profiles could be grouped into distinct clusters based on movement intensity and basketball efficiency statistics, and to identify the key performance indicators (KPIs) associated with different contextual factors, including close and large-margin quarters. By combining external load demands with match-related statistics, the present study aimed to provide a more comprehensive understanding of the multidimensional characteristics of performance in youth basketball, in which physical, technical, tactical, and contextual components continuously interact throughout competition. It was hypothesized that contextual factors, particularly score differential, would influence the characteristics of the identified performance profiles, with close-score-margin quarters exhibiting different combinations of external-load and match-related performance characteristics than large-score-margin quarters. Furthermore, it was expected that the performance characteristics observed in U16 basketball would differ from those previously reported in older age categories and adult populations, reflecting the distinct developmental, physical, and tactical demands of youth basketball competition. The association between cluster membership and quarter outcome was explored as a secondary descriptive analysis, and no a priori hypothesis was formulated for this analysis.

## 2. Materials and Methods

### 2.1. Study Design

The present study employed a data-driven analytical approach to identify performance profiles in basketball using unsupervised machine learning techniques. The objective was to detect latent groupings of team-quarter observations based on external load demands and match-related statistics without predefined labels.

The dataset comprised 19 official matches from the Final 8 phase of the Greek U16 Basketball Championship. Although the Final 8 represented the highest level of national competition for this age category, participating teams were not assumed to be homogeneous in competitive standard, and qualification did not necessarily indicate that all players performed at an elite level. Match-related box-score statistics and derived performance indicators were recorded using Sport Scout observational analysis software (Sport Scout STA Ver. 3.2, SportScout Group, Thessaloniki, Greece) and systematically documented in Microsoft Excel (Microsoft Inc., Washington, DC, USA) for data organization and subsequent statistical analysis. External-load demands were monitored using inertial measurement units (KINEXON Sports & Media GmbH, Munich, Germany). During each match, all players were continuously monitored from the beginning of the warm-up until the end of the match. However, external-load variables were quantified only during active on-court playing time. Periods of inactivity, including warm-up activities, time-outs, substitutions, bench time, and rest intervals between quarters and halftime, were excluded from the analysis. Following each match, all external load variables were processed and computed using the proprietary KINEXON performance analysis software (version 12.0; KINEXON Sports & Media GmbH, Munich, Germany). Permission to monitor the official matches was obtained from all participating teams and from the Hellenic Basketball Federation (H.B.F./E.O.K.). All players and coaching staff were informed about the study procedures, requirements, potential benefits, and possible risks associated with participation. Participation was entirely voluntary, and participants were informed that they could withdraw from the study at any time without penalty or any effect on their participation in the competition. Participants were additionally informed that the monitoring procedures were designed to minimize interference with match play and competitive performance. Written informed consent was obtained from each participant’s parent or legal guardian, and written assent was obtained from the participants before data collection, along with approval from both the participating teams and the Hellenic Basketball Federation. The study was conducted in accordance with the ethical principles of the Declaration of Helsinki (2024 revision). Ethical approval was obtained from the Ethics Committee of the Department of Physical Education and Sport Science, Democritus University of Thrace (Protocol No. DUTH/EHDE/38826/966/31 January 2025). All personal identifiers were removed before data analysis, and the anonymized dataset was securely stored and accessed only by the research team in accordance with institutional ethical requirements.

The complete dataset included match-related statistics and external load variables for all four quarters of each match. Initially, the dataset consisted of 132 team-quarter observations from matches in which the winner of the quarter prevailed by either a large or a small score difference. Before statistical analysis, 15 team-quarter observations were excluded, including eight quarter observations that ended in a tie and seven quarters with incomplete external load data due to missing recordings from one player. Consequently, the final dataset comprised 117 team-quarter observations for the subsequent analyses.

External load and basketball performance data were available for all individual players participating in each quarter. Cumulative external-load measures and team-level count variables, including points, rebounds, and assists, were aggregated as sums. In contrast, intensity-related metrics and shooting percentage variables were aggregated as averages. An unsupervised clustering framework was implemented to explore latent structures within the dataset. Specifically, the clustering analysis aimed to determine whether team-quarter observations could be grouped into distinct performance profiles based on movement intensity, physical load, and basketball efficiency variables. The clustering procedure was applied separately to team-quarter observations from quarters with large and small score differences, allowing examination of performance patterns under different competitive conditions. Following cluster identification, statistical analyses were conducted to examine differences between clusters and to evaluate the relationship between cluster membership and quarter outcomes. Because multiple quarter observations originated from the same teams, quarter observations were not statistically independent. Consequently, the results should be interpreted as exploratory and descriptive rather than as population-level inferences.

### 2.2. Data

#### 2.2.1. Match-Related Statistics Acquisition and Calculation

The following standard match-related statistics were recorded: field goals made (FGM), field goals attempted (FGA), 2-point shots made (2pt Made), 2-point shots attempted (2pt Att), 3-point shots made (3pt Made), 3-point shots attempted (3pt Att), free throws made (FT Made), free-throws attempted (FT Att), offensive rebounds (Of Reb), defensive rebounds (Def Reb), total rebounds (Total Reb), assists (AST), drawn fouls (Draw Fouls), fouls committed (Fouls), turnovers (TO), steals (ST), blocked shots (Blocks), points scored (Points), and points per possession (PPP). The analysis also included advanced composite basketball metrics such as effective field goal percentage (eFG%), offensive rating (OFFRTG), assist-to-turnover ratio (AST/TO), defensive rebound percentage (DREB%), turnover percentage (TO%), possessions (POSS), and performance index rating (PIR).

eFG% provides a pace-independent measure of overall shooting efficiency by accounting for the added value of three-point field goals. The metric is calculated as eFG% = [(FGM + 0.5 × 3pt Made)/FGA] × 100 [14]. OFFRTG expresses the number of points scored per 100 possessions. The calculation formula is as follows: OFFRTG = Points/POSS × 100. Ball possessions (POSS) were calculated with the following formula: POSS = FGA + (0.454 × FT Att) + TO − Of Reb [14]. A possession was defined as a sequence of play ending in a made field goal, a set of free throws, a turnover, or another event resulting in a change of possession. TO% estimates the percentage of team possessions that end in a turnover and is calculated as follows: TO% = (TO ÷ (FGA + (0.454 × FT Att) + TO)) × 100. AST/TO measures a team’s playmaking efficiency by dividing total assists by total turnovers, indicating the number of assists recorded per turnover. A higher AST/TO can indicate better ball control: AST/TO = Assists ÷ Turnovers. DREB% is the percentage of available defensive rebounds a team obtains, calculated as: Def Reb/(Def Reb + Opponent Of Reb) × 100. Higher percentages indicate superior defensive rebounding. Performance Index Rating (PIR) sums all positive player contributions (points, rebounds, assists, steals, blocks, fouls drawn) and subtracts negative ones (missed shots, turnovers, fouls committed) to provide a composite measure of overall team performance during each quarter. The formula used for the calculation of the metric was: PIR = (Points + Total Rebounds + Assists + Steals + Blocks + Fouls Drawn) − (Missed Field Goals + Missed Free Throws + TO + Blocked Shots + Fouls Committed).

Effective rotation size (ERS) represents the effective number of players reflected by the distribution of active playing time within each team-quarter observation. Higher ERS values indicate a more even and less concentrated distribution of playing time, whereas lower values indicate that playing time was concentrated among fewer players [37].

#### 2.2.2. Reliability of Match-Related Statistics

All match-related statistics were coded by a professional basketball coach with a degree in sport science and more than 10 years of experience in basketball performance analysis. To assess intra-observer reliability, the observer independently re-coded five randomly selected matches four weeks after the initial coding. Agreement between the two coding occasions was quantified using Cohen’s κ, which ranged from 0.91 to 0.95 across the recorded variables, indicating almost perfect agreement [38,39].

#### 2.2.3. External Load Monitoring

Before each match, each player wore a KINEXON Perform inertial measurement unit (IMU; KINEXON Sports & Media GmbH, Munich, Germany), which was housed in a custom leather pouch and secured with a clip to the waistband of the playing uniform [40]. The unit was positioned over the posterior pelvis, approximately at the level of the iliac crests, and aligned with the sagittal midline, in accordance with previously described procedures [40,41]. Across all matches, microsensor data were continuously recorded during official competition and downloaded following each match for further analysis using proprietary KINEXON software (version 12.0; KINEXON Sports & Media GmbH, Munich, Germany). The IMU device included a 3-axis accelerometer (±16 G; sampled at 100 Hz), a 3-axis gyroscope (±4000 deg/s; sampled at 200 Hz), and a 3-axis magnetometer (±16 μT; sampled at 100 Hz).

Accumulated acceleration load (AAL), termed Player Load in the device software, was calculated from sample-to-sample changes in acceleration across the three orthogonal axes and accumulated over active playing time. Specifically, AAL was calculated as the sum of the square root of the squared changes in acceleration across the *x*-, *y*-, and *z*-axes, divided by 100 [40,42,43], and was expressed in arbitrary units (AU). Previously reported coefficients of variation range from 0.91% to 1.05% within devices and from 1.02% to 1.90% between devices [40,42]. AAL+ was calculated as the sum of the AAL accumulated within the three highest manufacturer-defined intensity zones.

Mechanical load (ML) was calculated automatically by the proprietary software as a weighted sum of instantaneous acceleration and deceleration samples in the horizontal (*x* and *y*) planes across four acceleration and four deceleration categories. Mechanical intensity was calculated as mechanical load divided by active on-court playing time and was expressed in arbitrary units per minute (AU·min^−1^). Jump load (JL) was calculated for each jump as:

JL=m×g×h

where *m* represents body mass in kilograms, *g* represents gravitational acceleration in meters per second squared, and *h* represents estimated vertical displacement in meters. Jump load was summed across all jumps performed during active playing time. Jumps were categorized according to the 30 cm threshold as jumps below 30 cm (U30 cm) and jumps above 30 cm (O30 cm).

Distance was categorized into three speed zones: low speed (0–10.8 km·h^−1^), high speed (>10.8–18.72 km·h^−1^), and very high speed (>18.72 km·h^−1^). These thresholds were comparable to those previously applied in basketball research [42]. Physio Load was calculated automatically by the proprietary software using distance covered, body mass, and a manufacturer-defined sport-specific scaling factor. The software internally expressed distance in miles and body mass in pounds for this calculation. Where applicable, relative external-load variables were calculated by dividing the accumulated value by active on-court playing time and were expressed per minute [42].

Average live playing time was calculated as the mean duration of the active on-court segments used to aggregate the player-level external-load data for each team-quarter observation and was expressed in minutes.

#### 2.2.4. Datasets

For each eligible team-quarter observation, the quarter score differential was calculated as the absolute difference between the points scored by the observed team and the opposing team during that quarter. The distribution of quarter score differentials was examined, and the median value of 5 points was used to stratify the observations into two score-difference categories. The large-score-difference dataset comprised 55 team-quarter observations from quarters with a score differential of ≥6 points, whereas the small-score-difference dataset comprised 62 observations from quarters with a score differential of 1–5 points. This median-based threshold yielded two similarly sized datasets for separate category-specific clustering analyses. Following the exclusions described in Section 2.1, complete data were available for all variables included in the analyses.

Because the study employed an exploratory unsupervised clustering approach and did not test a predefined effect, no conventional a priori power analysis was performed. The analysis included all 117 eligible team-quarter observations, distributed across the two similarly sized datasets. The internal coherence of the identified clustering solutions and their sensitivity to centroid initialization were evaluated using multiple complementary internal-validation criteria and repeated clustering analyses. However, these procedures do not establish population-level sample adequacy, stability under resampling, or reproducibility in an independent sample. Accordingly, the identified profiles should be regarded as exploratory and require evaluation in larger independent datasets.

### 2.3. Unsupervised Learning

K-means clustering was applied separately to the large-score-difference and small-score-difference datasets to identify exploratory quarter-level performance profiles among the team-quarter observations. Separate clustering analyses were conducted because the study aimed to characterize performance profiles within each score context rather than to derive a single solution across all observations.

K-means was selected because all variables entered into the clustering analysis were continuous and standardized, and the analytical objective was to partition the observations into a small number of compact, mutually exclusive, and readily interpretable profiles. As a centroid-based partitioning method, k-means assigns each observation to the cluster with the nearest centroid under a Euclidean-distance framework, thereby facilitating the practical characterization of the resulting profiles according to differences across multiple external-load and match-related performance indicators.

K-means was preferred over hierarchical and density-based alternatives because these approaches were less closely aligned with the specific analytical objective of the present study. Hierarchical clustering is particularly useful when the objective is to examine nested relationships among observations or to derive a dendrogram representing multiple levels of grouping. Density-based methods are particularly advantageous when the objective is to identify irregularly shaped clusters and distinguish dense regions from isolated noise observations. The present analysis did not aim to identify nested structures, irregular cluster geometries, or noise points; instead, it sought a parsimonious, non-overlapping, and practically interpretable partition of the team-quarter observations. Accordingly, k-means was considered appropriate for the exploratory and practically oriented aims of the study. This methodological choice was also consistent with recent research in indoor team sports in which k-means was used to identify interpretable profiles from standardized multidimensional external-load data [44].

Before clustering, all continuous variables were standardized as *z*-scores, with a mean of 0 and a standard deviation of 1. Standardization was performed to prevent variables measured on larger numerical scales from exerting a disproportionate influence on the Euclidean distances used by the clustering algorithm. No preliminary feature-selection or dimensionality-reduction procedure was applied, and all available external-load and match-related performance variables were included in the clustering analyses.

Candidate solutions ranging from two to ten clusters (*k* = 2–10) were evaluated using four complementary internal-validation criteria. The Elbow method was used to examine changes in the within-cluster sum of squares across candidate solutions. The Silhouette coefficient was used to assess the cohesion of observations within their assigned clusters relative to their separation from other clusters. The Calinski–Harabasz Index was used to evaluate the ratio of between-cluster to within-cluster dispersion, whereas the Davies–Bouldin Index was used to assess the similarity between clusters based on their within-cluster dispersion and between-cluster separation. The number of clusters was selected through the combined interpretation of these criteria, together with consideration of solution parsimony and practical interpretability. The number of clusters retained for each score-context dataset is reported in Section 3.

For each dataset, k-means clustering was implemented using 100 centroid initializations (n_init = 100) and a fixed random seed (random_state = 42). The retained solution corresponded to the initialization that produced the lowest within-cluster inertia.

Sensitivity of the cluster assignments to centroid initialization was additionally examined using 100 repeated clustering solutions. Consistency among the resulting cluster assignments was quantified using the Adjusted Rand Index (ARI), with values closer to 1 indicating greater agreement between solutions. This procedure evaluated the dependence of the clustering solution on centroid initialization; it should not be interpreted as an assessment of stability under resampling or as evidence that the same profiles would necessarily be reproduced in an independent sample.

Finally, t-distributed stochastic neighbor embedding (t-SNE) was applied exclusively for visualization by projecting the standardized multidimensional data into two dimensions. The t-SNE coordinates were not used as inputs to the k-means algorithm, to determine the number of clusters, or as an independent criterion for validating cluster quality.

### 2.4. Statistical Analysis

Descriptive statistics were calculated for all variables and are presented as mean ± standard deviation (SD). Prior to inferential analyses, the distribution of continuous variables was assessed using the Shapiro–Wilk test. Because unequal variances were anticipated across several variables, differences between clusters were assessed using Welch’s independent-samples *t*-tests, which do not assume equal variances.

Given the large number of variables examined, the resulting *p*-values were adjusted for multiple comparisons using the Benjamini–Hochberg False Discovery Rate (FDR) procedure. Both the original (*p*) and adjusted (pFDR) values are reported. The Benjamini–Hochberg procedure was selected because it controls the false discovery rate while maintaining greater statistical power than more conservative family-wise error corrections, making it appropriate for exploratory multivariable analyses. Effect sizes were quantified using Cohen’s d and interpreted according to conventional thresholds as small (≈0.20), medium (≈0.50), and large (≥0.80).

Because the same variables were used both to derive and subsequently characterize the clusters, the between-cluster comparisons were interpreted as descriptive summaries of the identified performance profiles rather than as independent confirmatory tests of cluster validity. To examine the association between cluster membership and quarter outcome (win/loss), contingency-table analyses were performed. Pearson’s chi-square test was used when expected cell frequencies satisfied the required assumptions; otherwise, Fisher’s exact test was applied. Effect size was quantified using Cramer’s V.

All statistical analyses were performed in Python (version 3.11) using NumPy (version 2.3.5) and Pandas (version 2.3.3) for data management, scikit-learn (version 1.7.2) for machine learning analyses, SciPy (version 1.16.3) for statistical testing, and statsmodels (version 0.14.5) for multiple-comparison correction using the Benjamini–Hochberg FDR procedure. For analyses involving multiple simultaneous comparisons, statistical significance was evaluated using FDR-adjusted *p*-values (pFDR < 0.05). For all other analyses, the significance level was set at *p* < 0.05.

## 3. Results

Before clustering, pooled descriptive statistics were calculated for the complete dataset (*N* = 117 team-quarter observations). These pooled statistics were used solely to characterize the analytical sample; clustering was subsequently performed separately within the large-score-difference and small-score-difference datasets. Descriptive statistics for the external-load and locomotor variables are presented in Table 1.

Across the complete dataset, team-quarter observations included a mean total distance of 7254.39 ± 1242.03 m, an accumulated acceleration load of 1131.02 ± 183.35 AU, and 66.26 ± 16.39 jumps. The corresponding relative values were 83.62 ± 10.01 m·min^−1^, 13.08 ± 1.61 AU·min^−1^, and 0.74 ± 0.18 jumps·min^−1^, respectively.

The corresponding pooled descriptive statistics for match-related performance variables are presented in Table 2. Across the complete dataset, team-quarter observations included a mean of 14.98 ± 5.24 points, an offensive rating of 81.56 ± 31.92 points per 100 possessions, 0.82 ± 0.32 points per possession, an effective field-goal percentage of 40.00 ± 17.00%, and a Performance Index Rating of 15.70 ± 10.13.

Subsequent clustering analyses were conducted separately within the large-score-difference and small-score-difference datasets to identify context-specific performance profiles.

### 3.1. Large Score Differences

#### 3.1.1. Determination of the Optimal Number of Clusters

**Figure 1:**
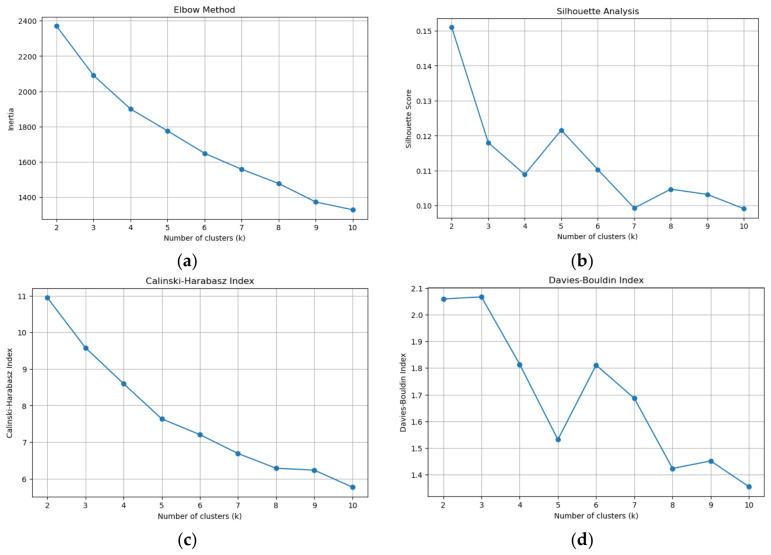
Internal-validation results for candidate clustering solutions in the large-score-difference dataset: (**a**) within-cluster inertia evaluated using the Elbow method, (**b**) Silhouette coefficient, (**c**) Calinski–Harabasz Index, and (**d**) Davies–Bouldin Index. Based on the combined interpretation of the four criteria, together with solution parsimony and practical interpretability, a two-cluster solution (*k* = 2) was retained.

The Silhouette coefficient and Calinski–Harabasz Index reached their highest values for the two-cluster solution (k = 2; Figure 1). The Elbow plot also showed progressively smaller reductions in within-cluster inertia as the number of clusters increased. Although the Davies–Bouldin Index did not identify k = 2 as the unique optimum, the combined interpretation of the internal-validation criteria, together with considerations of parsimony and practical interpretability, supported the retention of a two-cluster solution.

#### 3.1.2. Visualization of Cluster Structure

**Figure 2:**
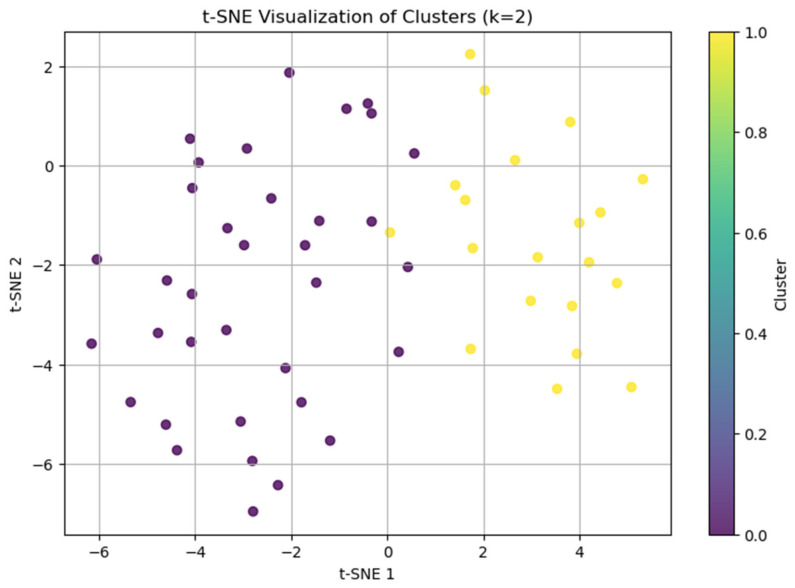
Two-dimensional t-SNE representation of the cluster assignments in the large-score-difference dataset. Each point represents a team-quarter observation, and colors indicate k-means cluster membership. The projection was used solely for visualization and did not contribute to cluster derivation or validation.

The two-dimensional t-SNE projection demonstrated a distinguishable spatial organization of the two identified clusters (Figure 2). As t-SNE was used exclusively for visualization purposes, this representation should be interpreted as a graphical illustration of the clustering solution rather than as an independent validation of cluster quality.

Cluster stability was evaluated by repeating the k-means algorithm across 100 different random initializations. The mean ARI across the resulting clustering solutions was 0.965 (range = 0.548–1.000), indicating a high degree of agreement between clustering solutions and supporting the stability of the identified cluster structure.

#### 3.1.3. Cluster Profiles and Statistical Comparisons

The two-cluster solution identified two distinct quarter performance profiles in matches with large score differences (Appendix A, Table A1). Overall, the higher-performance cluster was characterized by consistently greater external load and basketball performance values than the lower-performance cluster. The variables contributing most prominently to cluster differentiation included external load measures such as jump load, total distance covered, distance covered in the 10.8–18.72 km·h^−1^ and >18.72 km·h^−1^ speed zones, physiological load, mechanical load, AAL, and jump load per minute. Among the basketball performance indicators, OFFRTG, PIR, points scored, assists, field goals attempted and made, total rebounds, two-point field goals attempted and made, steals, and assist-to-turnover ratio showed the greatest differences between the two performance profiles.

Statistical comparisons between clusters using Welch’s independent-samples *t*-tests, with Benjamini–Hochberg FDR correction for multiple comparisons, identified several variables that remained statistically significant after FDR adjustment (Table A1). Overall, the higher-performance cluster combined greater movement intensity with higher offensive output and efficiency and match-related performance indicators. Because these comparisons describe variables contributing to the identified cluster profiles, they should be interpreted as a descriptive characterization of the clustering solution rather than as independent validation of cluster separation.

#### 3.1.4. Cluster Membership and Quarter Outcome

The higher-performance cluster contained a larger proportion of team-quarter observations classified as wins than the lower-performance cluster (70.0% vs. 45.7%; Table 3). However, Pearson’s chi-square test indicated no statistically significant association between cluster membership and quarter outcome, χ^2^(1) = 2.13, *p* = 0.145, Cramer’s V = 0.20. Thus, the observed difference in win proportions was descriptive and did not provide evidence of an association between cluster membership and quarter outcome.

### 3.2. Small Score Differences

#### 3.2.1. Determination of the Optimal Number of Clusters

**Figure 3:**
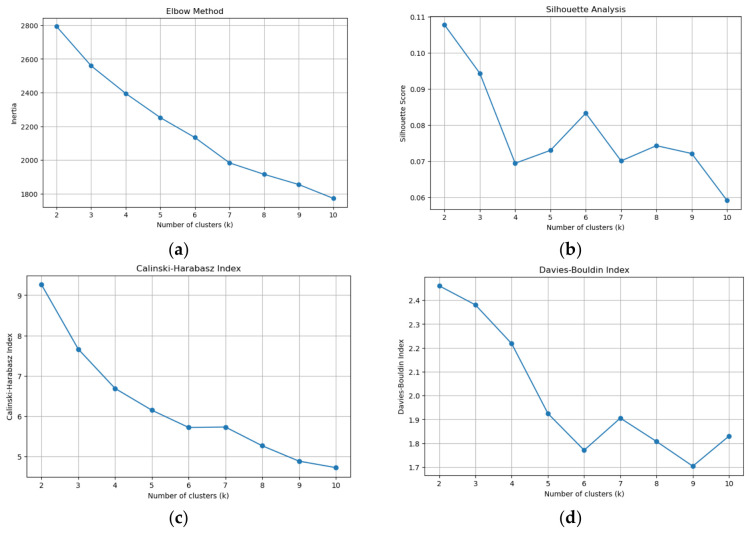
Internal-validation results for candidate clustering solutions in the small-score-difference dataset: (**a**) within-cluster inertia evaluated using the Elbow method, (**b**) Silhouette coefficient, (**c**) Calinski–Harabasz Index, and (**d**) Davies–Bouldin Index. Based on the combined interpretation of the four criteria, together with solution parsimony and practical interpretability, a two-cluster solution (*k* = 2) was retained.

The highest Silhouette coefficient and Calinski–Harabasz Index were observed for the two-cluster solution (*k* = 2; Figure 3). The Elbow plot also showed progressively smaller reductions in within-cluster inertia as the number of clusters increased. However, the maximum Silhouette coefficient was relatively low (0.108), indicating limited separation between the identified clusters, and the Davies–Bouldin Index favored some solutions with larger values of *k*. Based on the combined interpretation of the internal-validation criteria, together with considerations of parsimony and practical interpretability, a two-cluster solution was retained.

#### 3.2.2. Visualization of Cluster Structure

**Figure 4:**
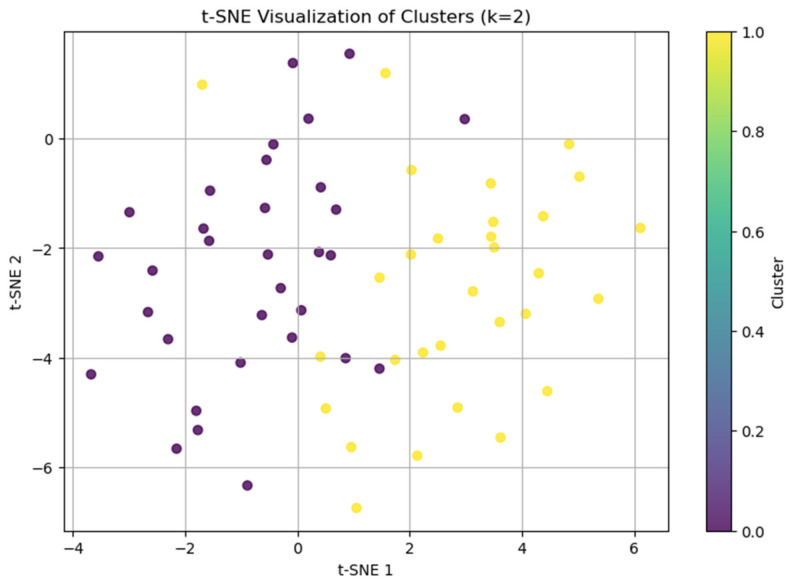
Two-dimensional t-SNE representation of the cluster assignments in the small-score-difference dataset. Each point represents a team-quarter observation, and colors indicate k-means cluster membership. The projection was used solely for visualization and did not contribute to cluster derivation or validation.

Cluster stability was evaluated by repeating the k-means algorithm across 100 different random initializations. The mean Adjusted Rand Index (ARI) across the resulting clustering solutions was 0.948 (range = 0.873–1.000), indicating a high degree of agreement between clustering solutions and supporting the stability of the identified cluster structure. The two-dimensional t-SNE visualization (Figure 4) further illustrated the separation between the two identified performance profiles.

#### 3.2.3. Cluster Profiles and Statistical Comparisons

The two-cluster solution identified two distinct quarter performance profiles in matches with small score differences. Overall, the higher-performance cluster was characterized by consistently greater external load and basketball performance values than the lower-performance cluster. The largest differences between clusters were observed for external load variables, including total distance covered, mechanical load, accumulated acceleration load, physiological load, AAL+, and distances covered in high- and very-high-speed zones. In addition, several basketball performance indicators differentiated the clusters, particularly points scored, possessions, free throws made and attempted, PIR, and offensive rating. Collectively, these findings indicate that the higher-performance profile combined greater physical demands with improved offensive production and overall match effectiveness, whereas the lower-performance profile was characterized by reduced external load and lower offensive output.

Following adjustment for multiple comparisons using the Benjamini–Hochberg false discovery rate procedure (Appendix B, Table A2), the higher-performance cluster exhibited significantly greater values for several external load variables, including total distance, mechanical load, accumulated acceleration load, physiological load, AAL, jump load, and distances covered at higher movement intensities. Among basketball performance indicators, significant differences were observed for possessions, points scored, free throws made and attempted, and fouls drawn. Overall, the results indicate that quarters characterized by greater movement intensity were also associated with higher offensive productivity in closely contested matches. Because these comparisons describe variables contributing to the identified cluster profiles, they should be interpreted as a descriptive characterization of the clustering solution rather than as independent validation of cluster separation.

#### 3.2.4. Cluster Membership and Quarter Outcome

The higher-performance cluster exhibited a greater proportion of winning team-quarter observations than the lower-performance cluster (56.7% vs. 40.6%; Table 4). However, contingency-table analysis did not demonstrate a statistically significant association between cluster membership and quarter outcome (χ^2^(1) = 1.02, *p* = 0.313, Cramer’s V = 0.13). Therefore, although a descriptive difference in win distribution was observed, cluster membership was not significantly associated with quarter outcome in quarters with small score differences.

## 4. Discussion

The present study used an unsupervised machine-learning approach to identify team-quarter performance profiles in U16 basketball by integrating external-load and match-related performance variables. Clustering was conducted separately within the large- and small-score-difference contexts. Two-cluster solutions were retained in both datasets, but the variables showing the largest between-cluster differences varied descriptively between contexts. In the large-score-difference dataset, substantial differences were observed in both external-load and offensive-performance variables, whereas in the small-score-difference dataset, the largest differences were concentrated in accumulated external-load measures. Although the higher-performance clusters contained greater proportions of winning quarters in both contexts, cluster membership was not significantly associated with quarter outcome. Accordingly, the identified profiles should be interpreted as descriptive combinations of co-occurring physical and match-related characteristics rather than as validated profiles of competitive success.

Few studies have simultaneously examined external-load and match-related performance variables in basketball [45,46,47]. Previous research has generally reported weak associations between physical and technical–tactical measures [45,46,47], while attempts to predict basketball performance using training- and competition-load data aggregated across the seasonal microcycle have also shown a limited predictive relationship [48]. To the authors’ knowledge, the present study is the first to identify quarter-level performance profiles in a national-level U16 competition by integrating external-load and match-related variables within an unsupervised machine-learning framework. The following sections discuss the findings according to score-difference context.

### 4.1. U16 External Load Demands

To the authors’ knowledge, no published study has examined external-load demands during national-level U16 male basketball tournament competition using quarter-based analysis. The only available study that partially addresses this age category was conducted by Cabarkapa et al. [33], which compared U16 and U18 female basketball players in official matches and reported no statistically significant differences between the age groups. Compared with the U16 female cohort of Cabarkapa et al. [33], the present U16 male sample showed higher mean values for distance covered per minute (83.62 ± 10.01 vs. 68.4 ± 3.4 m·min^−1^), maximum speed (22.32 ± 1.51 vs. 20.8 ± 1.1 km·h^−1^), mean speed (5.02 ± 0.60 vs. 4.1 ± 0.2 km·h^−1^), jump frequency (0.74 ± 0.18 vs. 0.66 ± 0.1 jumps·min^−1^), and AAL per minute (13.08 ± 1.61 vs. 6.68 AU·min^−1^). Although the present findings are discussed in relation to the available literature, direct comparisons should be interpreted with caution because the only comparable study was conducted in U16 female basketball players, whereas the present investigation involved U16 male players. Sex-related differences in external load and match demands may influence performance profiles and therefore limit direct numerical comparisons between studies. The present sample also showed higher AAL-per-minute values (13.08 ± 1.61 AU·min^−1^) than those reported in U18 male players by Salazar et al. [49] (11.02–11.39 AU·min^−1^), professional players by Svilar et al. [50] (11.1 ± 2.0 AU·min^−1^), and semiprofessional players by Conte et al. [51] (11.6 ± 1.5 AU·min^−1^). In contrast, jump frequency in the present study was substantially lower than the values reported for U18 players by Salazar et al. (1.55–1.67 jumps/min) [49] and for professional basketball players by Svilar et al. (1.11 ± 0.53 jumps/min) [50]. Similarly, the relative total distance covered in the present study (83.62 ± 10.01 m/min) was considerably lower than values previously reported for male professional players (133.1 ± 1.0 m/min) and college players (94.7 ± 9.2 m/min) [52,53]. These findings may indicate that, although the present U16 players covered a shorter relative distance and performed fewer jumping actions than older, more experienced basketball players, they were exposed to greater acceleration-based loads during competition. Since AAL represents a cumulative load across triaxial movement directions, elevated values may reflect a greater density of accelerations, decelerations, and rapid changes of direction rather than increased locomotor volume alone. These cross-study differences may reflect variation in age, sex, movement economy, playing style, possession structure, or match format; however, these factors were not directly assessed in the present study.

### 4.2. Large Score Difference Quarters

In quarters characterized by large score differences, two performance profiles were identified. The largest standardized between-cluster differences were observed for PIR (|d| = 2.04), assists (|d| = 1.91), and points scored (|d| = 1.76). Several other match-related performance variables, including OFFRTG, points per possession, field goals made, assist-to-turnover ratio, steals, and two-point field goals made, also differed between the profiles. Large standardized differences were also observed for several relative external-load variables, including mean speed and total distance covered per minute (both |*d*| = 1.56), distance covered over 18.72 km·h^−1^ per minute (|*d*| = 1.55), AAL+ per minute (|*d*| = 1.32), AAL per minute (|*d*| = 1.30), and distance covered at 10.8–18.72 km·h^−1^ per minute (|*d*| = 1.29). Thus, the profiles differed in both offensive output and movement-related characteristics, rather than being separated primarily by one performance domain.

Previous research has consistently associated steals with match outcome because they terminate opposing possessions and create transition opportunities that may lead to high-quality scoring attempts [17,54,55]. Their value extends beyond preventing a scoring opportunity, as steals can generate additional possessions and initiate transition play, which typically lead to high-quality, efficient shots [17,56]. Assists and field goals have also been associated with successful basketball performance, reflecting the importance of effective ball distribution and shot conversion [17,57,58]. Previous studies have also identified OFFRTG and PIR as box-score indicators associated with team success and match outcome [17,59,60]. One possible explanation is that greater movement intensity may facilitate transition opportunities and fast-break situations, which are generally associated with more efficient scoring opportunities because the opposing defense has less time to organize [61]. However, transition frequency and defensive organization were not directly measured in the present study. Previous research has similarly reported associations between mechanical activity, heart-rate responses, and offensive efficiency in junior basketball players [62]. Although the higher-performance cluster contained a greater proportion of winning quarters (70.0% vs. 45.7%), cluster membership was not significantly associated with quarter outcome. The identified profile should therefore be interpreted as a combination of co-occurring physical and offensive characteristics rather than as a validated profile of competitive success. Quarter outcome is likely influenced by additional factors that were not captured in the clustering analysis, including defensive performance [54], match management [63], situational decision-making [64], and the evolving score context.

### 4.3. Small Score Difference Quarters

Evidence regarding quarter-level relationships between external load and match-related performance variables, and their association with quarter outcomes, remains limited in U16 basketball. The largest standardized between-cluster differences were concentrated in external-load variables, including total distance covered, mechanical load, accumulated acceleration load, Physio Load, and AAL+ (|*d*| = 1.98–2.35). Among the non-external-load variables, estimated possessions (POSS) and effective rotation size (ERS) showed large standardized between-cluster differences (|*d*| = 0.98 and 1.09, respectively), whereas differences in most technical-efficiency variables were less pronounced. Medium standardized between-cluster differences were observed for average live playing time, fouls committed, free throws attempted, jumps under 30 cm, jumps under 30 cm per minute, mechanical intensity, AST/TO, distance covered over 18.72 km·h^−1^ per minute, PIR, FGA, and FGM (|*d*| = 0.50–0.77). Overall, the largest between-cluster differences in small-score-difference quarters were concentrated in external-load variables. The number of possessions is closely related to match pace, with a greater number of possessions generally reflecting a faster pace of play [65].

In small-score-difference quarters, the largest between-cluster differences were concentrated in accumulated external-load measures, whereas differences in most offensive-efficiency indicators were smaller. In such contexts, teams may rely more heavily on structured offensive systems, controlled pacing, and risk-minimization strategies, as they prioritize tactical execution and game control, aiming to reduce turnovers and enhance defensive intensity [66], thereby reducing variability in technical performance indicators across possessions. In interpreting the results, it should not be overlooked that variables such as Average Live Playing Time, FT, Steals, Fouls, FGM, 2pt, AST/TO, and jumps above 30 cm also contributed to cluster separation and demonstrated moderate-to-large effect sizes (|d| = 0.50–0.80). Although adjustments in game pace may be an important strategy for increasing offensive opportunities or enhancing defensive effectiveness, variations in team play styles and individual player characteristics suggest that faster or slower pacing does not necessarily guarantee competitive success [67]. Although the higher-performance cluster contained a greater proportion of winning quarters (56.7% vs. 40.6%), cluster membership was not significantly associated with quarter outcome. Thus, greater movement demands distinguished the clusters but did not clearly correspond to superior offensive efficiency or competitive success.

### 4.4. Comparison Between Quarter Contexts

A key contribution of the present study lies in comparing performance profiles across different score contexts. Across both score contexts, two-cluster solutions were identified, but the variables showing the largest between-cluster differences varied by context. In large-score-difference quarters, substantial differences were observed in both external-load and offensive-performance variables. In small-score-difference quarters, the largest differences were concentrated in accumulated external-load measures, whereas differences in offensive-efficiency measures were generally smaller. These descriptive contrasts suggest that the composition of the identified profiles varied across score contexts, although no direct statistical comparison between contexts was performed. In large-score-difference quarters, the identified profiles showed clearer differences in match-related and offensive-efficiency indicators. Conversely, in small-score-difference quarters, the largest between-cluster differences were concentrated mainly in external-load variables, whereas differences in offensive-efficiency indicators were less pronounced. Previous research has identified differences in external load measures across match quarters and has demonstrated positive, moderate-to-large relationships with box score statistics [68]. These findings imply that a faster game pace increases movement demands and scoring opportunities. By contrast, a recent study of professional men’s basketball reported only small-to-trivial associations between basketball performance metrics and match loads [47]. One possible explanation is that, at the professional level, decision-making and technical and tactical proficiency may weaken the direct relationship between external-load measures and match-related performance indicators. These findings support the conceptualization of basketball as a complex, dynamic system in which performance outcomes are shaped by the interaction of physical, technical, and contextual factors rather than by linear cause-and-effect relationships.

### 4.5. Practical Applications, Limitations, and Future Directions

The main practical implication of the present findings is that external-load data should not be interpreted in isolation. Within the identified profiles, quarters with greater movement demands also showed higher values in several offensive-performance indicators. However, cluster membership was not significantly associated with quarter outcome. Coaches should therefore interpret physical-load measures together with technical-performance indicators and match context, rather than regarding greater physical output as evidence of more successful performance.

Several limitations should be considered. The analysis included 117 team-quarter observations from a single U16 Final 8 competition, which limits the extent to which the identified profiles can be generalized. In addition, multiple quarters originated from the same teams and matches and were therefore not statistically independent. The clusters were also generated from the same variables subsequently used to describe them. As a result, the post-clustering comparisons characterize the profiles but do not provide independent validation of the clustering solution. The inclusion of numerous correlated raw, relative, and derived variables may also have increased the influence of overlapping performance dimensions. Furthermore, dividing score differential according to the sample median reduced a continuous contextual variable to two broad categories. Consistency across different k-means initializations indicates that the algorithm repeatedly produced similar cluster assignments from different starting points, but it does not establish that the same profiles would emerge in an independent sample. Defensive pressure, shot quality, spatial organization, tactical behavior, and player maturation were not directly assessed.

Future studies should examine larger independent datasets across multiple seasons, competitions, and age groups. Analyses should account for the nesting of quarters within teams and matches and test whether the identified profiles remain stable under different variable selections, score-context definitions, resampling procedures, and clustering algorithms. The inclusion of player-tracking and tactical data would also provide a more complete account of how physical, technical, and spatial factors interact in youth basketball.

## 5. Conclusions

The present study investigated quarter performance profiles in elite U16 basketball using an unsupervised ML approach integrating external load variables and match-related performance indicators across different score contexts. Two-cluster solutions were retained in both datasets, although the variables showing the largest between-cluster differences varied according to score context. In large-score-difference quarters, substantial differences were observed in both relative external-load and match-related performance variables, whereas in small-score-difference quarters, the largest differences were concentrated in accumulated external-load measures. In the large-score-difference dataset, the higher-performance profile also showed higher offensive output and efficiency, whereas corresponding differences in the small-score-difference dataset were generally less pronounced. These findings suggest that movement intensity and external load represent important characteristics of basketball performance, whereas overall match performance is likely influenced by a broader interaction of contextual, tactical, and defensive factors. Although higher-performance clusters exhibited greater winning proportions, no statistically significant association was observed between cluster membership and quarter outcome. Overall, the results highlight the importance of adopting an integrated approach to basketball performance analysis that combines physical load metrics with technical performance indicators. Furthermore, the application of unsupervised ML techniques provided a useful framework for identifying latent performance structures within complex datasets, offering a valuable tool for performance analysis in team sports. Future research should extend this approach by incorporating larger datasets and additional contextual variables, such as defensive behavior, spatial positioning, and tactical organization, evaluating alternative clustering algorithms, and validating the identified performance profiles using independent datasets. The integration of physical, technical, tactical, and spatial data may provide a more comprehensive understanding of basketball performance.

## Figures and Tables

**Table 1:** Pooled descriptive statistics for external-load and locomotor variables across the 117 team-quarter observations.

Variables	Mean	SD
Maximum speed (km·h^−1^)	22.32	1.51
Mean speed (km·h^−1^)	5.02	0.60
Total distance (m)	7254.39	1242.03
Distance at 0–10.8 km·h^−1^ (m)	4863.13	882.88
Distance at 10.8–18.72 km·h^−1^ (m)	2092.44	507.90
Distance at >18.72 km·h^−1^ (m)	297.58	141.92
Accumulated acceleration load (AU)	1131.02	183.35
Accumulated acceleration load (AU·min^−1^)	13.08	1.61
Total jumps (n)	66.26	16.39
Jumps (O30 cm)	36.20	10.40
Jumps (U30 cm)	30.06	8.42
Jumps/min	0.74	0.18
Jumps/min (O30 cm)	0.40	0.12
Jumps/min (U30 cm)	0.34	0.10
Total distance (m·min^−1^)	83.62	10.01
Distance at 0–10.8 km·h^−1^ (m·min^−1^)	55.58	6.80
Distance at 10.8–18.72 km·h^−1^ (m·min^−1^)	24.14	4.48
Distance at >18.72 km·h^−1^ (m·min^−1^)	3.54	1.76

**Table 2:** Pooled descriptive statistics for match-related performance variables across the 117 team-quarter observations.

Variables	Mean	SD
Points	14.98	5.24
Free throws made (FT Made)	2.68	2.02
Free throws attempted (FT Att)	4.70	2.78
Free-throw percentage (FT%)	54.95	28.95
Two-point field goals made (2pt Made)	4.09	2.00
Two-point field goals attempted (2pt Att)	9.71	3.33
Two-point field-goal percentage (2pt%)	43.60	18.57
Three-point field goals made (3pt Made)	1.21	1.15
Three-point field goals attempted (3pt Att)	5.32	2.34
Three-point field-goal percentage (3pt%)	23.04	21.83
Fouls committed	4.31	1.69
Fouls drawn	4.41	1.68
Defensive rebounds (Def Reb)	6.82	2.27
Offensive rebounds (Of Reb)	2.73	1.99
Total rebounds (Total Reb)	9.55	3.28
Assists (AST)	3.53	2.02
Steals (ST)	2.52	1.66
Blocks	0.90	1.11
Turnovers (TO)	4.13	2.17
Performance Index Rating (PIR)	15.70	10.13
Defensive rebound percentage (DREB%)	72	16
Effective field-goal percentage (eFG%)	40	17
Assist-to-turnover ratio (AST/TO)	1.21	1.18
Offensive rating (OFFRTG)	81.56	31.92
Field goals attempted (FGA)	14.95	3.27
Field goals made (FGM)	5.30	2.23
Estimated possessions (POSS)	18.42	2.58
Turnover percentage (TO%)	22	11
Points per possession (PPP)	0.82	0.32

**Table 3:** Distribution of quarter outcomes according to cluster membership in the large-score-difference dataset.

Cluster	Loss (%)	Win (%)
Lower performance cluster	54.3%	45.7%
Higher performance cluster	30.0%	70.0%

Note. Pearson’s χ^2^(1) = 2.13, *p* = 0.145, Cramer’s V = 0.20. The minimum expected cell frequency was 9.09; therefore, the assumptions of Pearson’s chi-square test were satisfied.

**Table 4:** Distribution of team-quarter outcomes according to cluster membership for quarters with small score differences.

Cluster	Loss (%)	Win (%)
Lower performance cluster	59.4%	40.6%
Higher performance cluster	43.3%	56.7%

Note. Pearson’s χ^2^(1) = 1.02, *p* = 0.313, Cramer’s V = 0.13. The minimum expected cell frequency was 14.52; therefore, the assumptions of Pearson’s chi-square test were satisfied.

## Data Availability

The data are available from the corresponding Author following a reasonable request.

## Appendix A

**Table A1:** Cluster characteristics and descriptive between-cluster comparisons in the large-score-difference dataset.

Variable	Lower-Performance Cluster (Mean ± SD)	Higher-Performance Cluster (Mean ± SD)	Welch’s t	*p*	pFDR	Cohen’s d
Performance Index Rating (PIR)	10.51 ± 10.49	29.65 ± 7.01	−8.09	<0.001	<0.001	−2.04
Assists (AST)	2.60 ± 1.77	5.75 ± 1.41	−7.25	<0.001	<0.001	−1.91
Points	13.11 ± 5.31	21.65 ± 3.84	−6.87	<0.001	<0.001	−1.76
Mean speed (km·h^−1^)	4.81 ± 0.59	5.61 ± 0.34	−6.4	<0.001	<0.001	−1.56
Total distance (m·min^−1^)	80.15 ± 9.86	93.54 ± 5.65	−6.4	<0.001	<0.001	−1.56
Accumulated acceleration load (AU·min^−1^)	12.63 ± 1.66	14.51 ± 0.91	−5.4	<0.001	<0.001	−1.3
AAL+ (AU·min^−1^)	8.66 ± 1.46	10.37 ± 0.95	−5.28	<0.001	<0.001	−1.32
Distance at 10.8–18.72 km·h^−1^ (m·min^−1^)	22.87 ± 4.86	28.41 ± 3.03	−5.2	<0.001	<0.001	−1.29
Points per possession (PPP)	0.69 ± 0.29	1.22 ± 0.41	−5.1	<0.001	<0.001	−1.56
Offensive rating (OFFRTG)	69.33 ± 29.11	122.13 ± 40.88	−5.09	<0.001	<0.001	−1.56
Distance over 18.72 km·h^−1^ (m·min^−1^)	2.93 ± 1.20	5.34 ± 2.03	−4.84	<0.001	<0.001	−1.55
Distance at 0–10.8 km·h^−1^ (m·min^−1^)	54.70 ± 5.65	59.78 ± 3.46	−4.13	<0.001	<0.001	−1.02
Distance over 18.72 km·h^−1^ (m)	240.49 ± 97.36	400.00 ± 145.58	−4.37	<0.001	<0.001	−1.36
Maximum speed (km·h^−1^)	21.78 ± 1.47	23.12 ± 1.03	−3.94	<0.001	<0.001	−1.01
Assist-to-turnover ratio (AST/TO)	0.72 ± 0.62	2.44 ± 1.78	−4.18	<0.001	0.001	−1.46
Jump load (J·min^−1^)	177.88 ± 40.51	231.26 ± 52.45	−3.93	<0.001	0.001	−1.18
Two-point field goals attempted (2pt Att)	8.43 ± 2.62	11.90 ± 3.46	−3.89	<0.001	0.001	−1.18
Field goals made (FGM)	4.51 ± 2.09	7.25 ± 2.90	−3.7	<0.001	0.002	−1.13
Distance at 10.8–18.72 km·h^−1^ (m)	1883.86 ± 412.87	2262.20 ± 376.85	−3.46	0.001	0.003	−0.95
Steals (ST)	2.17 ± 1.36	3.85 ± 1.93	−3.44	0.002	0.005	−1.06
Fouls drawn	4.60 ± 1.68	3.30 ± 1.26	3.25	0.002	0.005	0.84
Defensive rebound percentage (DREB%)	64 ± 18	78 ± 13	−3.23	0.002	0.005	−0.82
Jumps over 30 cm (O30 cm; jumps·min^−1^)	0.39 ± 0.11	0.49 ± 0.12	−3.27	0.002	0.005	−0.93
Jumps under 30 cm (U30 cm; jumps·min^−1^)	0.32 ± 0.09	0.41 ± 0.11	−3.12	0.004	0.008	−0.93
Field goals attempted (FGA)	13.86 ± 2.06	16.70 ± 3.71	−3.16	0.004	0.008	−1.03
Two-point field goals made (2pt Made)	3.40 ± 1.82	5.25 ± 2.40	−2.99	0.005	0.011	−0.9
AAL+ (AU)	732.39 ± 141.88	839.20 ± 126.51	−2.88	0.006	0.012	−0.78
Defensive rebounds (Def Reb)	6.09 ± 2.27	7.75 ± 1.97	−2.85	0.007	0.012	−0.77
Jump load (J)	15,658.95 ± 3865.08	18,715.71 ± 4055.77	−2.73	0.009	0.017	−0.78
Effective field-goal percentage (eFG%)	36 ± 15	54 ± 26	−2.73	0.011	0.019	−0.88
Jumps over 30 cm (O30 cm)	33.97 ± 9.12	40.30 ± 8.87	−2.52	0.016	0.026	−0.7
Total rebounds (Total Reb)	8.54 ± 2.70	11.10 ± 3.99	−2.55	0.016	0.026	−0.79
Physio Load	1241.70 ± 244.50	1390.39 ± 208.26	−2.39	0.021	0.033	−0.64
Three-point field goals made (3pt Made)	1.11 ± 1.11	2.00 ± 1.45	−2.37	0.024	0.037	−0.71
Total distance (m)	6741.34 ± 1308.03	7468.20 ± 1088.80	−2.21	0.032	0.048	−0.59
Starting score difference at quarter onset (points)	−2.63 ± 15.23	5.90 ± 14.29	−2.08	0.044	0.062	−0.57
Free throws attempted (FT Att)	5.29 ± 2.60	3.80 ± 2.53	2.08	0.044	0.062	0.58
Mechanical intensity (AU·min^−1^)	233.58 ± 53.04	261.32 ± 45.14	−2.05	0.046	0.063	−0.55
Accumulated acceleration load (AU)	1061.96 ± 204.84	1154.23 ± 168.47	−1.8	0.078	0.104	−0.48
Jumps under 30 cm (U30 cm)	27.60 ± 7.84	31.15 ± 7.00	−1.73	0.091	0.118	−0.47
Turnovers (TO)	4.60 ± 2.10	3.55 ± 2.28	1.69	0.1	0.127	0.48
Turnover percentage (TO%)	25 ± 10	20 ± 13	1.44	0.16	0.199	0.43
Effective rotation size (ERS)	6.55 ± 1.09	6.92 ± 0.92	−1.34	0.185	0.224	−0.36
Free throws made (FT Made)	2.97 ± 1.96	2.25 ± 1.92	1.33	0.191	0.225	0.37
Offensive rebounds (Of Reb)	2.46 ± 1.22	3.35 ± 3.01	−1.27	0.218	0.252	−0.44
Average live playing time (min)	11.03 ± 2.92	10.19 ± 2.11	1.23	0.224	0.253	0.32
Mechanical load	2471.93 ± 507.25	2580.08 ± 388.16	−0.89	0.38	0.42	−0.23
Distance at 0–10.8 km·h^−1^ (m)	4615.94 ± 950.40	4804.70 ± 740.06	−0.82	0.417	0.452	−0.21
Blocks	0.80 ± 1.28	0.95 ± 1.00	−0.48	0.632	0.657	−0.13
Fouls committed	4.03 ± 1.67	4.25 ± 1.59	−0.49	0.628	0.657	−0.13
Estimated possessions (POSS)	18.33 ± 1.95	18.57 ± 3.23	−0.31	0.759	0.773	−0.1
Three-point field goals attempted (3pt Att)	5.43 ± 2.37	5.30 ± 1.75	0.23	0.819	0.819	0.06

Note. Values are presented as mean ± standard deviation (SD). Differences between clusters were evaluated using Welch’s independent-samples *t*-tests. *p*-values were adjusted for multiple comparisons using the Benjamini–Hochberg false discovery rate (FDR) procedure. Cohen’s d is reported as the standardized effect size. Starting score difference was calculated at quarter onset as the observed team’s cumulative score minus the opposing team’s cumulative score; positive values indicate that the team was leading, negative values that it was trailing, and 0 that the score was tied.

## Appendix B

**Table A2:** Cluster characteristics and descriptive between-cluster comparisons in the small-score-difference database.

Variable	Lower-Performance Cluster (Mean ± SD)	Higher-Performance Cluster (Mean ± SD)	Welch’s t	*p*	pFDR	Cohen’s d
Total distance (m)	6597.94 ± 881.25	8410.63 ± 634.98	−9.33	<0.001	<0.001	−2.35
Mechanical load	2404.79 ± 303.94	3050.47 ± 248.53	−9.18	<0.001	<0.001	−2.32
Accumulated acceleration load (AU)	1043.83 ± 140.52	1289.13 ± 79.72	−8.52	<0.001	<0.001	−2.13
Physio Load	1223.97 ± 171.17	1565.64 ± 158.19	−8.17	<0.001	<0.001	−2.07
AAL+ (AU)	719.03 ± 103.93	887.20 ± 58.08	−7.93	<0.001	<0.001	−1.98
Distance at 10.8–18.72 km·h^−1^ (m)	1822.91 ± 283.99	2510.13 ± 573.25	−5.92	<0.001	<0.001	−1.53
Distance over 18.72 km·h^−1^ (m)	212.47 ± 98.09	386.70 ± 136.38	−5.74	<0.001	<0.001	−1.47
Jump load (J)	14,856.53 ± 3036.83	19,287.46 ± 3682.97	−5.15	<0.001	<0.001	−1.32
Distance at 0–10.8 km·h^−1^ (m)	4561.47 ± 681.34	5512.23 ± 786.33	−5.07	<0.001	<0.001	−1.30
Maximum speed (km·h^−1^)	21.62 ± 1.43	23.17 ± 1.32	−4.44	<0.001	<0.001	−1.13
Jumps over 30 cm (O30 cm)	30.66 ± 7.32	41.97 ± 12.02	−4.44	<0.001	<0.001	−1.15
Effective rotation size (ERS)	6.34 ± 0.89	7.34 ± 0.94	−4.28	<0.001	<0.001	−1.09
Fouls drawn	3.94 ± 1.72	5.43 ± 1.28	−3.90	<0.001	0.001	−0.98
Estimated possessions (POSS)	17.26 ± 2.08	19.66 ± 2.81	−3.81	<0.001	0.001	−0.98
Points	12.28 ± 3.08	15.60 ± 3.83	−3.74	<0.001	0.001	−0.96
Free throws made (FT Made)	1.91 ± 1.42	3.47 ± 2.40	−3.09	0.003	0.011	−0.80
Average live playing time (min)	10.72 ± 2.11	12.34 ± 2.06	−3.04	0.004	0.011	−0.77
Fouls committed	3.94 ± 1.81	5.07 ± 1.48	−2.69	0.009	0.027	−0.68
Jumps under 30 cm (U30 cm; jumps·min^−1^)	0.36 ± 0.11	0.30 ± 0.06	2.61	0.012	0.033	0.65
Free throws attempted (FT Att)	3.78 ± 2.09	5.60 ± 3.42	−2.51	0.016	0.041	−0.65
Mechanical intensity (AU·min^−1^)	229.13 ± 40.69	252.95 ± 40.41	−2.31	0.024	0.060	−0.59
Jumps under 30 cm (U30 cm)	28.59 ± 6.80	33.77 ± 10.39	−2.30	0.025	0.060	−0.59
Assist-to-turnover ratio (AST/TO)	0.85 ± 0.61	1.35 ± 1.11	−2.18	0.035	0.078	−0.56
Distance over 18.72 km·h^−1^ (m·min^−1^)	2.90 ± 1.53	3.73 ± 1.55	−2.14	0.037	0.079	−0.54
Performance Index Rating (PIR)	12.62 ± 5.95	15.73 ± 6.16	−2.02	0.048	0.100	−0.51
Field goals attempted (FGA)	14.16 ± 2.94	15.90 ± 3.82	−2.01	0.050	0.100	−0.51
Offensive rating (OFFRTG)	71.40 ± 16.79	79.62 ± 16.99	−1.92	0.060	0.108	−0.49
Field goals made (FGM)	4.72 ± 1.55	5.53 ± 1.74	−1.94	0.057	0.108	−0.50
Points per possession (PPP)	0.71 ± 0.17	0.80 ± 0.17	−1.92	0.059	0.108	−0.49
Steals (ST)	1.94 ± 1.34	2.67 ± 1.67	−1.89	0.064	0.111	−0.48
Two-point field goals attempted (2pt Att)	9.00 ± 2.77	10.50 ± 3.77	−1.78	0.081	0.136	−0.46
Assists (AST)	2.97 ± 1.53	3.73 ± 2.05	−1.65	0.104	0.169	−0.42
Distance at 0–10.8 km·h^−1^ (m·min^−1^)	56.21 ± 6.30	53.15 ± 8.90	1.55	0.126	0.199	0.40
Two-point field goals made (2pt Made)	3.78 ± 1.70	4.47 ± 1.91	−1.49	0.141	0.216	−0.38
Blocks	1.12 ± 1.16	0.73 ± 0.94	1.46	0.148	0.221	0.37
Turnover percentage (TO%)	23 ± 10	20 ± 10	1.21	0.233	0.336	0.31
AAL+ (AU·min^−1^)	9.01 ± 1.36	8.66 ± 1.02	1.13	0.264	0.371	0.28
Accumulated acceleration load (AU·min^−1^)	13.05 ± 1.71	12.69 ± 1.31	0.94	0.351	0.480	0.24
Effective field-goal percentage (eFG%)	37 ± 11	39 ± 12	−0.74	0.461	0.614	−0.19
Distance at 10.8–18.72 km·h^−1^ (m·min^−1^)	23.18 ± 4.29	23.78 ± 3.45	−0.61	0.545	0.708	−0.15
Three-point field goals made (3pt Made)	0.94 ± 0.98	1.07 ± 0.98	−0.52	0.606	0.769	−0.13
Defensive rebounds (Def Reb)	7.06 ± 2.55	6.80 ± 2.01	0.45	0.653	0.809	0.11
Defensive rebound percentage (DREB%)	75 ± 15	74 ± 15	0.41	0.681	0.823	0.11
Three-point field goals attempted (3pt Att)	5.16 ± 2.44	5.40 ± 2.66	−0.38	0.709	0.831	−0.10
Jump load (J·min^−1^)	184.36 ± 37.95	188.29 ± 51.62	−0.34	0.735	0.831	−0.09
Total rebounds (Total Reb)	9.75 ± 3.78	9.47 ± 2.47	0.35	0.727	0.831	0.09
Turnovers (TO)	4.12 ± 2.28	3.97 ± 2.09	0.28	0.777	0.859	0.07
Jumps over 30 cm (O30 cm; jumps·min^−1^)	0.38 ± 0.09	0.39 ± 0.13	−0.21	0.834	0.890	−0.05
Starting score difference at quarter onset (points)	0.72 ± 11.71	0.13 ± 10.85	0.20	0.839	0.890	0.05
Offensive rebounds (Of Reb)	2.69 ± 2.29	2.67 ± 1.52	0.04	0.966	0.966	0.01
Mean speed (km·h^−1^)	4.95 ± 0.64	4.94 ± 0.47	0.06	0.955	0.966	0.01
Total distance (m·min^−1^)	82.43 ± 10.68	82.32 ± 7.86	0.05	0.963	0.966	0.01

Note. Values are presented as mean ± standard deviation (SD). Differences between clusters were evaluated using Welch’s independent-samples *t*-tests. *p*-values were adjusted for multiple comparisons using the Benjamini–Hochberg false discovery rate (FDR) procedure. Cohen’s d is reported as the standardized effect size. Starting score difference was calculated at quarter onset as the observed team’s cumulative score minus the opposing team’s cumulative score; positive values indicate that the team was leading, negative values that it was trailing, and 0 that the score was tied.

## References

[1] Ribeiro J., Silva P., Duarte R., Davids K., Garganta J. (2017). Team Sports Performance Analysed Through the Lens of Social Network Theory: Implications for Research and Practice. Sports Med..

[2] Wright C., Atkins S., Jones B., Todd J. (2013). The Role of Performance Analysts within the Coaching Process: Performance Analysts Survey ‘The Role of Performance Analysts in Elite Football Club Settings’. Int. J. Perform. Anal. Sport.

[3] Gabbett T.J. (2016). The Training—Injury Prevention Paradox: Should Athletes Be Training Smarter and Harder?. Br. J. Sports Med..

[4] Lord F., Pyne D.B., Welvaert M., Mara J.K. (2020). Methods of Performance Analysis in Team Invasion Sports: A Systematic Review. J. Sports Sci..

[5] Greenberg W., Clubb J. (2021). Why ‘Best Practice’ Is Not Always Best in Sport. Br. J. Sports Med..

[6] McLean S., Robertson S., Salmon P.M. (2025). Complexity and Systems Thinking in Sport. J. Sports Sci..

[7] McLean S., Read G.J.M., Hulme A., Dodd K., Gorman A.D., Solomon C., Salmon P.M. (2019). Beyond the Tip of the Iceberg: Using Systems Archetypes to Understand Common and Recurring Issues in Sports Coaching. Front. Sports Act. Living.

[8] Travassos B., Araújo D., Duarte R., McGarry T. (2012). Spatiotemporal Coordination Behaviors in Futsal (Indoor Football) Are Guided by Informational Game Constraints. Hum. Mov. Sci..

[9] McGarry T. (2009). Applied and Theoretical Perspectives of Performance Analysis in Sport: Scientific Issues and Challenges. Int. J. Perform. Anal. Sport.

[10] McGarry T., Anderson D.I., Wallace S.A., Hughes M.D., Franks I.M. (2002). Sport Competition as a Dynamical Self-Organizing System. J. Sports Sci..

[11] Reed D., Hughes M. (2006). An Exploration of Team Sport as a Dynamical System. Int. J. Perform. Anal. Sport.

[12] Salmon P.M., McLean S. (2020). Complexity in the Beautiful Game: Implications for Football Research and Practice. Sci. Med. Footb..

[13] Stojanović E., Stojiljković N., Scanlan A.T., Dalbo V.J., Berkelmans D.M., Milanović Z. (2018). The Activity Demands and Physiological Responses Encountered During Basketball Match-Play: A Systematic Review. Sports Med..

[14] Kubatko J., Oliver D., Pelton K., Rosenbaum D.T. (2007). A Starting Point for Analyzing Basketball Statistics. J. Quant. Anal. Sports.

[15] Skinner B., Guy S.J. (2015). A Method for Using Player Tracking Data in Basketball to Learn Player Skills and Predict Team Performance. PLoS ONE.

[16] Plakias S., Kokkotis C., Pantazis D., Tsatalas T. (2024). Comparative Analysis of Key Performance Indicators in Euroleague and National Basketball Leagues. J. Phys. Educ. Sport.

[17] Foteinakis P.F., Pavlidou S.P. (2025). Game-Related Performance Metrics Differentiating Winning and Losing Teams in the Basketball Champions League. J. Phys. Educ..

[18] Foteinakis P.F., Kokkotis C., Karamousalidis G., Avloniti A., Pavlidou S., Zaras N., Stampoulis T., Pantazis D., Aggelakis P., Balampanos D. (2025). From Data to Decisions: Using Explainable Machine Learning to Predict EuroLeague Basketball Outcomes. Appl. Sci..

[19] Fox J.L., Scanlan A.T., Stanton R. (2017). A Review of Player Monitoring Approaches in Basketball: Current Trends and Future Directions. J. Strength Cond. Res..

[20] Svilar L., Jukic I. (2018). Load Monitoring System in Top-Level Basketball Team: Relationship between External and Internal Training Load. Kinesiology.

[21] Petway A.J., Freitas T.T., Calleja-González J., Medina Leal D., Alcaraz P.E. (2020). Training Load and Match-Play Demands in Basketball Based on Competition Level: A Systematic Review. PLoS ONE.

[22] Fox J.L., Stanton R., Sargent C., O’Grady C.J., Scanlan A.T. (2020). The Impact of Contextual Factors on Game Demands in Starting, Semiprofessional, Male Basketball Players. Int. J. Sports Physiol. Perform..

[23] Wang J. (2024). Predictive Analysis of NBA Game Outcomes through Machine Learning. Proceedings of the 6th International Conference on Machine Learning and Machine Intelligence, Chongqing, China, 27–29 October 2023.

[24] Liang Z., Gao Y., Wang J., Liu Z. (2025). Data-Driven Insights into Basketball Performance: Unveiling the Impact of Advanced Analytics on Player and Team Efficiency. Mol. Cell. Biomech..

[25] Sansone P., Alonso Perez Chao E., Li F., Gasperi L., Gómez-Ruano M.A., Conte D. (2025). Contextual Factors Influencing Basketball Training and Competition Demands: A Systematic Review. Int. J. Sports Med..

[26] Scanlan A.T., Tucker P.S., Dascombe B.J., Berkelmans D.M., Hiskens M.I., Dalbo V.J. (2015). Fluctuations in Activity Demands Across Game Quarters in Professional and Semiprofessional Male Basketball. J. Strength Cond. Res..

[27] Angel Gómez M., Lorenzo A., Sampaio J., Ibáñez S.J., Ortega E. (2008). Game-Related Statistics That Discriminated Winning and Losing Teams from the Spanish Men’s Professional Basketball Teams. Coll. Antropol..

[28] Csataljay G., James N., Hughes M., Dancs H. (2012). Performance Differences between Winning and Losing Basketball Teams during Close, Balanced and Unbalanced Quarters. J. Hum. Sport Exerc..

[29] Pérez-Chao E., Gómez M., Scanlan A., Ribas C., Trapero J., Lorenzo A. (2023). Influence of Game and Quarter Results on External Peak Demands during Games in Under-18 Years, Male Basketball Players. Biol. Sport.

[30] Piñar M.I., García D., Mancha-Triguero D., Ibáñez S.J. (2022). Effect of Situational and Individual Factors on Training Load and Game Performance in Liga Femenina 2 Basketball Female Players. Appl. Sci..

[31] Bustamante-Sánchez Á., Alonso-Perez-Chao E., Portes R., Leite N. (2025). Internal and External Loads in U16 Women’s Basketball Players Participating in U18 Training Sessions: A Case Study. Appl. Sci..

[32] Gonçalves G., Neta P., Ribeiro J., Guimaraes E. (2025). Internal and External Loads during Formal Training and Competition, Physical Capacities, and Technical Skills in Youth Basketball: A Comparison between Starters and Rotation Players. J. Hum. Kinet..

[33] Cabarkapa D.V., Cabarkapa D., Nagy D., Balogh L., Laczko T., Ratgeber L. (2025). Game vs. Practice Differences in External Load in U16 and U18 Women’s Basketball Players. Sports.

[34] Zhang S., Li M., Qin S., Xing W., Zhai Z., Wang X. (2025). Tracking Key Metrics: Fluctuations in External and Internal Load across Game Quarters in Collegiate Basketball Players. BMC Sports Sci. Med. Rehabil..

[35] Garcia F., Molina R., Alonso Perez-Chao E., Li M., Zhang S., Salazar H. (2025). Inertial Movement Demands Comparison between Winning and Losing Quarters in Youth Basketball Players. Kinesiology.

[36] Robertson P.S. (2020). Man & Machine: Adaptive Tools for the Contemporary Performance Analyst. J. Sports Sci..

[37] Hill M.O. (1973). Diversity and Evenness: A Unifying Notation and Its Consequences. Ecology.

[38] Cohen J. (1960). A Coefficient of Agreement for Nominal Scales. Educ. Psychol. Meas..

[39] Altman D.G. (1991). Practical Statistics for Medical Research.

[40] Pantazis D., Kokkotis C., Zaras N., Balampanos D., Avloniti A., Stampoulis T., Foteinakis P.F., Frazis Christou P., Papoulias G., Aggelakis P. (2026). Individualized Physical Performance Metrics in 3 × 3 Basketball Games Using Match-Play Data. Appl. Sci..

[41] Barrett S., Midgley A., Lovell R. (2014). PlayerLoad^TM^: Reliability, Convergent Validity, and Influence of Unit Position during Treadmill Running. Int. J. Sports Physiol. Perform..

[42] Pantazis D., Stampoulis T., Balampanos D., Avloniti A., Kokkotis C., Aggelakis P., Protopapa M., Draganidis D., Emmanouilidou M., Retzepis N.-O. (2025). Comparing Workloads Among Different Age Groups in Official Masters’ Basketball Matches: Implications for Physical Activity. Appl. Sci..

[43] Balampanos D., Pantazis D., Kokkotis C., Avloniti A., Stampoulis T., Aggelakis P., Nedeltsos E., Kaltsos G., Protopapa M., Retzepis N.-O. (2026). Physical Activity During Official Match Play in Female Masters Basketball Players: An Accelerometry-Based Study. Sports.

[44] Kokkotis C., Kansizoglou I., Pantazis D., Avloniti A., Balampanos D., Foteinakis P., Stampoulis T., Protopapa M., Dendrinos A., Aggelakis P. (2026). Data-Driven Reduction of External Load Variables in Indoor Team Sports Using Local Positioning System. J. Funct. Morphol. Kinesiol..

[45] Vučković I., Rátgéber L., Nagy D., Čabarkapa D., Mikić M., Kukić F. (2026). Load Dynamics in Basketball: Insights from Wins and Losses. Montenegrin J. Sports Sci. Med..

[46] Fox J.L., Stanton R., O’Grady C.J., Teramoto M., Sargent C., Scanlan A.T. (2022). Are Acute Player Workloads Associated with In-Game Performance in Basketball?. Biol. Sport.

[47] García F., Fernández D., Martín L. (2022). Relationship Between Game Load and Player’s Performance in Professional Basketball. Int. J. Sports Physiol. Perform..

[48] Li G., Shang L., Qin S., Yu H. (2024). The Impact of Internal and External Loads on Player Performance in Chinese Basketball Association. BMC Sports Sci. Med. Rehabil..

[49] Salazar H., Garcia F., Svilar L., Castellano J. (2021). Physical Demands in Three Different Basketball Competitions Played By the Same Under-18 Players. Montenegrin J. Sports Sci. Med..

[50] Svilar L., Castellano J., Jukic I. (2019). Comparison of 5vs5 Training Games and Match-Play Using Microsensor Technology in Elite Basketball. J. Strength Cond. Res..

[51] Conte D., Kamarauskas P., Ferioli D., Scanlan A.T., Kamandulis S., Paulauskas H., Lukonaitienė I. (2021). Workload and Well-Being across Games Played on Consecutive Days during the In-Season Phase in Basketball Players. J. Sports Med. Phys. Fit..

[52] Koyama T., Nishikawa J., Yaguchi K., Irino T., Rikukawa A. (2024). A Comparison of the Physical Demands Generated by Playing Different Opponents in Basketball Friendly Matches. Biol. Sport.

[53] Scanlan A., Dascombe B., Reaburn P. (2011). A Comparison of the Activity Demands of Elite and Sub-Elite Australian Men’s Basketball Competition. J. Sports Sci..

[54] Buyukcelebi H., Sahin F.N., Acak M., Uysal H.Ş., Sari C., Erkan D., Yatak S., Karayigit R. (2024). Changes in Defensive Variables Determining Success in the NBA over the Last 10 Years. Appl. Sci..

[55] Leicht A.S., Gómez M.A., Woods C.T. (2017). Explaining Match Outcome During The Men’s Basketball Tournament at The Olympic Games. J. Sports Sci. Med..

[56] Matulaitis K., Bietkis T. (2021). Prediction of Offensive Possession Ends in Elite Basketball Teams. Int. J. Environ. Res. Public Health.

[57] Mikołajec K., Banyś D., Żurowska-Cegielska J., Zawartka M., Gryko K. (2021). How to Win the Basketball Euroleague? Game Performance Determining Sports Results During 2003–2016 Matches. J. Hum. Kinet..

[58] García J., Ibáñez S.J., De Santos R.M., Leite N., Sampaio J. (2013). Identifying Basketball Performance Indicators in Regular Season and Playoff Games. J. Hum. Kinet..

[59] Kamitsis A., Politis K. (2025). Performance Indicators for Qualification to the Playoffs and the Final Four in Euroleague 2000–2022. J. Sports Anal..

[60] Sampaio J., McGarry T., Calleja-González J., Jiménez Sáiz S., Schelling i del Alcázar X., Balciunas M. (2015). Exploring Game Performance in the National Basketball Association Using Player Tracking Data. PLoS ONE.

[61] Conte D., Favero T., Niederhausen M., Capranica L., Tessitore A. (2017). Determinants of the Effectiveness of Fast Break Actions in Elite and Sub-Elite Italian Men’s Basketball Games. Biol. Sport.

[62] Bazanov B., Rannama I. (2017). The Relationship between Physiological and Mechanical Load Indicators and Offensive Team Efficiency in Junior Male Basketball. J. Hum. Sport Exerc..

[63] Qiu M., Zhang S., Yi Q., Zhou C., Zhang M. (2024). The Influence of “Momentum” on the Game Outcome While Controlling for Game Types in Basketball. Front. Psychol..

[64] Rösch D., Schultz F., Höner O. (2021). Decision-Making Skills in Youth Basketball Players: Diagnostic and External Validation of a Video-Based Assessment. Int. J. Environ. Res. Public Health.

[65] Sakalidis K.E., Pérez-Tejero J., Khudair M., Hettinga F.J. (2024). Ball Possessions and Game Rhythm in Basketball Games Involving Players with and without Intellectual Impairments. J. Intellect. Disabil. Res..

[66] Mandić R., Jakovljević S., Erčulj F., Štrumbelj E. (2019). Trends in NBA and Euroleague Basketball: Analysis and Comparison of Statistical Data from 2000 to 2017. PLoS ONE.

[67] Chen R., Zhang M., Xu X., Liu Y. (2025). Game-Related Statistics for Distinguishing Winning and Losing Teams in Olympic Basketball: The Impact of Game Pace. J. Sports Sci..

[68] Brown F.S.A., Fields J.B., Jagim A.R., Baker R.E., Jones M.T. (2024). Analysis of In-Season External Load and Sport Performance in Women’s Collegiate Basketball. J. Strength Cond. Res..

